# One Year Follow Up Efficacy of the Coping Power Universal and Its Relations with Teachers’ Occupational Stress

**DOI:** 10.3390/children8100832

**Published:** 2021-09-22

**Authors:** Valentina Levantini, Emanuela Ala, Iacopo Bertacchi, Giulia Cristoni, Sara Maggi, Gaelle Pontrandolfo, Monica Torsellini, John E. Lochman, Pietro Muratori

**Affiliations:** 1 IRCCS Stella Maris, Scientific Institute of Child Neurology and Psychiatry, 56100 Pisa, Italy; giulia_cristoni@hotmail.it (G.C.); sara.maggi@fsm.unipi.it (S.M.); pietro.muratori@fsm.unipi.it (P.M.); 2 ADHD Piemonte—Famiglie Associate, 10100 Torino, Italy; emiala@hotmail.com; 3 Associazione Mente Cognitiva, 55100 Lucca, Italy; iacopobertacchi@hotmail.com; 4 Delegazione Territoriale A.P.I.CI Piana di Lucca, Servizio Punto Handy, 55100 Lucca, Italy; gaelle.pontrandolfo@virgilio.it; 5 AIDAI Toscana, 50100 Firenze, Italy; monicatorsellini@yahoo.it; 6 Department of Psychology, The University of Alabama, Tuscaloosa, AL 35487, USA; jlochman@ua.edu

**Keywords:** internalizing, externalizing, prosocial, evidence-based programs, teachers’ stress

## Abstract

The coping power universal (CPU) is an evidence-based universal prevention program delivered by teachers, and completely integrated into the school agenda. Previous studies have shown its positive effects, though little is known about its longer-term effects, and no previous study has explored whether teachers’ occupational stress could influence the CPU efficacy. The current study aimed to explore the 1 year follow up of the CPU on students’ externalizing and internalizing problems and prosocial behavior, and the influence of baseline levels of teachers’ stress in a sample of 316 3rd graders and their teachers (*N* = 32). Results showed that the CPU led to positive effects, not attainable with the standard curriculum. Additionally, improvements in prosocial behavior persisted even one year after the conclusion of the program. However, improvements in internalizing and externalizing problems were not maintained at the follow up, highlighting the need to understand the factors influencing the CPU efficacy. In this regard, our findings showed that high levels of teachers’ occupational stress predicted poorer improvements following the CPU, and an increase in students’ difficulties at the follow-up assessment. Addressing teachers’ stress as part of prevention programs for students could boost their efficacy and yield more lasting results.

## 1. Introduction

The prevention of adverse outcomes and treatment of students’ psychopathology, along with the promotion of positive development, has become a priority for schools. In this perspective, there was an increase in the implementation of evidence-based programs (EBPs) in the school context. A vast scientific literature has shown that these programs positively influence the students’ social and emotional skills, reduce behavioral and emotional difficulties, and boost prosocial behavior and academic skills [1,2]. More importantly, such results are achieved without draining the already limited schools’ resources (e.g., time, money, available staff).

One of the most widespread EBPs is the coping power program (CPP). In its original form, developed by Lochman and Wells [3], the CPP is an EBP for children at risk for developing aggressive behavioral problems, usually delivered by a counselor or psychologist in a small group format and includes implementing different cognitive-behavioral techniques (e.g., token economy, goal setting, self-control techniques, and relaxation). More recently, the CPP was adapted as a universal prevention program, called the coping power universal (CPU), to prevent behavioral problems in all the students in a class [4], using the same principles and practices of the original CPP. However, the CPU is delivered by the teachers to classes of 20–25 students, and it is wholly integrated with the routine school activities. The change of setting made it necessary to make modifications to the original CPP activities (e.g., use of a storybook to guide the CPU activities).

A wealth of studies has recognized the CPU’s positive effects, including reducing behavioral problems, improving prosocial behavior, and academic skills [4,5,6,7,8], to the point that it was included in the Blueprints for Healthy Youth Development’s list of certified promising programs. However, to date, only a study has tested whether the positive effects of the CPU are maintained in the long term [8]. Despite the promising results of this previous work, more studies investigating whether the CPU can yield long-lasting effects are needed.

Moreover, no previous studies have investigated whether teachers’ characteristics are associated with reduced CPU effects. In this regard, an important factor that should be considered is teachers’ occupational stress. Studies have shown that levels of teachers’ stress had increased dramatically worldwide [9,10,11,12,13,14]. High levels of teachers’ occupational stress are related to numerous factors, including, but not limited to: managing students’ difficult behavior and conduct problems, excessive workload (at school and home), relational difficulties with colleagues, perceived lack of status [15]. Stress levels can influence the teachers’ ability to manage their classrooms and are usually associated with poorer job satisfaction and reduced teaching efficacy [9,16,17,18]. Moreover, when stress is persistently high, it can hinder teachers’ performance, affect their ways of interacting with students, contributing to a more stressful classroom climate [19], eventually leading to an increase in students’ problematic behavior [20]. It is, therefore, conceivable that teachers’ occupational stress might influence the effects of an EBP. A greater understanding of how teachers’ occupational stress relates to the program’s effects may help develop teachers’ training protocols and ongoing support efforts.

The current study aimed to test the 1-year follow-up effects of the CPU on students’ internalizing problems, externalizing problems, and prosocial behavior. It also aimed to test whether teachers’ occupational stress was associated with reduced CPU efficacy. This study tested these hypotheses in a sample independent from those used in our previous studies on the CPU.

## 2. Materials and Methods

Participants were 316 3rd grade students (54% female) and their classroom teachers (*N* = 32) attending public schools in Italy. Students’ age ranged from 8 to 9 years (mean age = 8.45 years; *SD* = 0.32); 87% of the students were Caucasian, 10% were Africans, and 3% were of other races.

The recruited classes were randomly assigned to either CPU intervention (*N* = 8) or the control condition (*N* = 8). The class was the unit for group assignment, and the random allocation sequence was computer-generated.

The assessment of students’ emotional and behavioral difficulties and prosocial behavior was conducted before the intervention (November 2017, T0), after the intervention (May 2018, T1), and approximately one year later (May 2019, T2). Before the intervention, we also assessed teachers’ occupational stress. All the teachers from the intervention classes attended an eight-hour training workshop in October 2017, and they delivered the intervention in their classes from December 2017 to April 2018.

The 32 classroom teachers were two males and 30 females, aged from 32 to 47 years. All teachers had completed a certified, university-based teacher education program in the past.

All parents signed a written informed consent form to let their children participate. All participating teachers provided consent. The study was conducted according to the guidelines of the Declaration of Helsinki and approved by the Institutional Review Board of the involved school district (PTOF, prot. 4, 13 September 2017).

### 2.1. Measures

Teachers completed the Italian version of the strengths and difficulties questionnaire (SDQ) [21,22] before the intervention, at the end of the intervention, and one year after its conclusion. The SDQ assessed the students’ emotional and behavioral difficulties, and prosocial behavior using 25 items, grouped into five subscales (i.e., emotional difficulties, conduct problems, hyperactivity symptoms, peer problems, and prosocial behavior). For the current study, along with the prosocial behavior and overall difficulties scores, we used the internalizing problems (i.e., emotional difficulties, peer problems) and externalizing problems (i.e., conduct problems, hyperactivity) composite scores, as they are deemed more appropriate with community samples [23]. The Cronbach’s alphas were 0.80 for overall difficulties, 0.82 for internalizing, 0.81 for externalizing, and 0.85 for prosocial behavior.

Teachers also completed a questionnaire about the stressful factors associated with the teaching profession—*Questionario sui Fattori Stressanti nell’Insegnamento* [24]. The questionnaire includes 30 items assessing different sources of stress teachers have to manage, including workload, relationships with colleagues and superiors, and the class environment (e.g., students’ challenging behaviors, large classes). The Cronbach’s alpha was 0.80 for this measure.

### 2.2. Intervention

The CPU includes 24 weekly sessions delivered by the school teachers. The CPU activities aim to improve students’ behavioral and cognitive abilities and emotion recognition, expression, and regulation. The CPU lessons focus on self-control, emotion awareness, awareness of the physiological arousal linked to emotions, and problem-solving skills. Each session lasted 45 min and involved revising the weekly goal sheets, the activities for the specific session, and the assignment of points, which were given for participating in the daily activities and achieving weekly goals. The CPU program was carried out during school hours as part of the daily school routine. The CPU intervention manual describes each lesson, though teachers were free to adapt the activities to the characteristics of their students. A more detailed description of the CPU activities is reported in previous studies, see for instance [4,8].

All the teachers from the intervention classes attended an eight-hour training workshop in October 2017, where they learned about the theoretical frame of the intervention, its experimental basis, and the CPU activities. Teachers in the CPU classes were deemed capable of intervening after attending the entire training provided by one of our researchers. Teachers also took part in monthly meetings, where they discussed and solved difficulties they had encountered during the implementation of the intervention. A school psychologist trained in the CPU model monitored the teachers’ adherence to the intervention using a checklist, monitoring whether the main lesson elements were delivered as intended. The checklists revealed that 87% of the elements of the CPU intervention sessions were delivered.

The classrooms assigned to the control group followed the standard curriculum activities provided in the Italian school context, which did not involve the exposure to EBPs promoting social and emotional skills.

### 2.3. Statistical Analyses

All the statistical tests were run on IBM SPSS Statistics for Windows, Version 23.0 (IBM Corp., Armonk, NY, USA). We tested the 1 year follow-up effects of the CPU on overall difficulties, externalizing problems, internalizing problems, and prosocial behavior with a series of linear mixed-effects models (MIXED) with full-information maximum likelihood (ML) estimation [25]. The analysis featured a three-level (measurement occasion within individuals within classes) random-intercept model to account for within-subject correlations and within-class correlations. Each model included subject and classrooms as random effects and group (CPU vs. control), time (T0, T1, T2), and their interaction as fixed effects, with a heterogeneous first-order autoregressive (ARH (1)) covariance structure. We did not include school variables as a random effect because we collected data from only two schools, which is not large enough for including these variables in the model. To further investigate the significant interactions, we explored the effect of time on overall difficulties, externalizing problems, internalizing problems, and prosocial behavior separately for the CPU and control groups. Bonferroni correction was applied to adjust for multiple comparisons.

Finally, we run a series of linear regressions to test whether the baseline levels of teachers’ stress predicted reduced CPU effects. Specifically, we used teachers’ stress at T0 as the independent variable and changes in students’ outcomes between T0 and T1, or T1 and T2 as the dependent variables. Changes in students’ outcomes were computed as residualized changes because it allowed adjusting for baseline differences [26]. The residualized change score is the difference between the observed score at post-intervention and the predicted score at post-intervention, where the pre-intervention measure was used to predict the post-intervention measure.

## 3. Results

Table 1 describes the two groups at T0, T1, and T2.

### 3.1. CPU Effects

#### 3.1.1. Overall Difficulties

Linear mixed models showed a significant effect of group, time, and group × time on students’ overall difficulties (see Table 2). Further analyses revealed a significant effect of time on overall difficulties in both the control (*F* = 5.21, *p* = 0.007) and CPU (*F* = 23.41, *p* < 0.001) groups. In the control group, overall difficulties at T1 were significantly higher than those at T0 (mean difference = 0.704, *p* = 0.016, *d* = 0.22), while no significant difference emerged between overall difficulties at T2 and T1 (mean difference = 0.195, *p* = 1.00, *d* = 0.08) and T0 (Mean difference = 0.899, *p* = 0.130, *d* *=* 0.25). In the CPU group, overall difficulties at T1 were significantly lower than those at T0 (mean difference = −1.624, *p* < 0.001, *d* *=* −0.53). However, overall difficulties at T2 were higher than those at T1 (mean difference = 1.33, *p* = 0.012, *d* *=* 0.25) and not statistically different from those at T0 (mean difference = −0.288, *p* = 1.00, *d* *=* −0.06).

#### 3.1.2. Externalizing Problems

Linear mixed models showed a significant effect of group, time, and group × time on students’ externalizing problems (see Table 2). Further analyses revealed a significant effect of time on externalizing problems in both the control (*F* = 9.93, *p* < 0.001) and CPU (*F* = 16.12, *p* < 0.001) groups. In the control group, externalizing problems at T1 (mean difference = 0.607, *p* < 0.001, *d* *=* 0.37) and T2 (mean difference = 0.629, *p* = 0.04, *d* *=* 0.32) were significantly higher than those at T0, while no significant difference emerged between externalizing problems at T1 and T2 (mean difference = 0.022, *p* = 1.00, *d* *=* 0.09). In the CPU group, externalizing problems at T1 were significantly lower than those at T0 (mean difference = −0.870, *p* < 0.001, *d* *=* −0.47). No significant difference emerged between externalizing problems at T2 and T1 (mean difference = 0.592, *p* = 0.132, *d* *=* 0.17) or T0 (mean difference = −0.277, *p* = 1.00, *d* *=* −0.09).

#### 3.1.3. Internalizing Problems

Linear mixed models showed a significant effect of group × time on students’ internalizing problems (see Table 2). Further analyses revealed a significant effect of time on internalizing problems in the CPU group (*F* = 15.53, *p* < 0.001) but not in the controls (*F* = 0.577, *p* = 0.56). In the CPU group, internalizing problems at T1 were significantly lower than those at T0 (mean difference = −0.764, *p* < 0.001, *d* = −0.41). Internalizing problems at T2 were higher than internalizing problems at T1 (mean difference = 0.742, *p* = 0.007, *d* *=* 0.023) and not significantly different from those at T0 (mean difference = −0.022, *p* = 1.00, *d* = −0.01).

#### 3.1.4. Prosocial Behavior

Linear mixed models showed a significant effect of group × time on students’ prosocial behavior (see Table 2). Further analyses revealed a significant effect of time on prosocial behavior in the CPU group (*F* = 20.48, *p* < 0.001) but not in the controls (*F* = 0.483, *p* = 0.62). In the CPU group, prosocial behavior scores at T1 were significantly higher than those at T0 (mean difference = 0.832, *p* < 0.001, *d* *=* 0.048), and prosocial behavior scores at T2 were significantly higher than T0 (mean difference = 0.649, *p* *=* 0.003, *d =* 27) and not statistically different from those at T1 (mean difference = −0.183, *p* = 0.850, *d* = −0.09).

### 3.2. Teachers’ Occupational Stress Influence on CPU Outcomes

Linear regressions showed that teachers’ stress at T0 predicted changes between T0 and T1 in overall difficulties (β = 0.147, *p* = 0.011, C.I. [0.012–0.089]) and internalizing problems (β = 0.188, *p* = 0.001, C.I. [0.015–0.061]). Namely, higher levels of teachers’ occupational stress were associated with fewer changes in students’ overall difficulties and internalizing problems following the CPU.

Moreover, teachers’ stress at T0 predicted changes between T1 and T2 in overall difficulties (β = −0.367, *p* < 0.001, C.I. [−0.309–−0.166]), externalizing problems (β = −0.378, *p* < 0.001, C.I. [−0.203–−0.112]), and internalizing problems (β = −0.248, *p* < 0.001, C.I. [−0.124–−0.045]). Specifically, higher levels of teachers’ stress at baseline with greater increase in overall difficulties, externalizing and internalizing problems between T1 and T2.

## 4. Discussion

Schools are fertile contexts for implementing EBPs to prevent the emergence of children’s emotional and behavioral difficulties and promote protective factors (e.g., social-emotional skills, pro-sociality), as they allow to reach large numbers of youths at the same time easily. Several EBPs were developed, and the CPU, based on Lochman and Wells’ CPP [3], is one of the most widely used in Italian schools. The current study aimed to test its 1-year follow up effects on primary students’ emotional and behavioral difficulties and prosocial behavior.

Results showed a decrease in students’ emotional and behavioral problems assessed with the SDQ at the end of the CPU (i.e., T1). Specifically, the internalizing problems, externalizing problems, and overall difficulties scores at T1 were significantly lower than the corresponding scores at T0. Additionally, the CPU was able to improve children’s prosocial behavior. These findings are consistent with previous studies showing the positive effects of the CPU on students’ outcomes. Indeed, previous studies found an improvement in externalizing and internalizing problems and prosocial behavior in primary school pupils, e.g., [4,8], and in internalizing problems and prosocial behavior in middle school students [5].

Findings pertaining to the effects of the CPU showed that the improvement in students’ prosocial behavior was preserved at the follow up. However, students’ emotional and behavioral difficulties appeared to increase between T1 and T2, though remaining slightly lower than the baseline assessment. Notably, in the control group, students’ emotional and behavioral difficulties increased over time and at the follow-up assessment were a little higher than the CPU group (see Table 1). Conversely, prosocial behavior in the control students did not change over time and was lower than students exposed to the CPU at both T1 and T2.

Our results suggest that the CPU can yield some positive effects, compared to the standard curriculum activities provided in the Italian school context. Additionally, some of the improvements persisted even one year after the conclusion of the program (i.e., prosocial behavior). However, improvements in internalizing and externalizing problems were not maintained at the follow up, highlighting the need to understand further the factors influencing the CPU efficacy. The CPU applies a whole-class token economy, in which the entire class gain a reward only if all the pupils reach their individual goal. Using this CBT technique might have induced the results related to prosocial behavior. The less optimistic results about internalizing and externalizing problems gives us good reasons to assume that the CPU could benefit from including specific activities addressing these difficulties. Overall, our results are consistent with studies showing that very few programs are actually able to reach considerable effects [27].

In this regard, the current study also aimed to test whether teachers’ baseline levels of occupational stress were associated with reduced improvements across time. Results showed that higher levels of stress predicted students’ overall difficulties and internalizing problems following the CPU. Moreover, higher stress predicted a greater increase in overall difficulties, externalizing, and internalizing problems between the post-intervention assessment and the follow up.

Teachers who experience high levels of occupational stress are less satisfied with their job, and this could influence their relationships with the students and their sense of self-efficacy [9,16,17,18]. Some teachers may find the CPU activities as an adjunctive source of stress and, therefore, be less motivated and less involved in their implementation, reducing the CPU effectiveness. The CPU does not target teachers’ occupational stress, though it is possible that addressing this issue (e.g., counseling, support therapy, and group meeting) might improve the intervention efficacy and help to reach more enduring positive effects. Future studies should further investigate the long-term effects of the CPU, the factors that can influence its effectiveness, and whether addressing teachers’ stress can boost the CPU efficacy.

The results of the current study need to be interpreted in light of some limitations. First, students’ outcomes were assessed with behavior ratings rather than direct observations. We do not have information about the percentage of students with neurodevelopmental disorders in the CPU or control groups. Moreover, the SDQ was completed by the same teachers who delivered the intervention. Since they underwent specific training about the CPU theoretical framework and objective, they might have been unwittingly influenced, especially in their post-treatment reports. Future studies should include multi-method approaches to assess students’ emotional, behavioral outcomes, and academic outcomes [28]. The sample size for the CPU teachers was small, limiting the interpretation and generalizability of the results about the influence of occupational stress. Future studies should also take into account the importance of teacher–class interactions [29] and the influence of classroom climate on behavioral problems [30].

## 5. Conclusions

The current study provides further evidence of the CPU efficacy. Indeed, results showed improvement in students’ outcomes following the CPU. More importantly, the improvement in prosocial behavior was maintained at the follow-up assessment. The latter is an encouraging and promising result since prosocial behavior is linked to several positive individual characteristics and outcomes, such as empathic skills, good peer relationships, and well-being [31,32]. Moreover, prosocial behavior is considered a protective factor for conduct problems and aggressive behavior [33,34].

However, our results also highlighted how difficult it might be to reach long-lasting effects following an EBP in the school context. Different factors can influence the EBPs implementation and effectiveness, including perceived staff and principal support, practitioners’ variables [35,36,37,38]. Our study suggests that teachers’ occupational stress might play a significant role in determining the EBP efficacy. Mindfulness-based interventions (MBI) could serve as adjunctive programs, along with the EBPs for students. The use of MBIs for school staff is rapidly increasing, and studies have shown that mindfulness has the potential to reduce stress and risk of burnout, improve teachers’ well-being and mental health, enhance job performance, and also improve classroom management, teacher–student relationships, and instructional strategies (for a review see [39]). It is possible that including MBIs for teachers as part of programs for students could benefit both the teachers and the students, and eventually boost the EBPs efficacy.

## Figures and Tables

**Table 1:** Descriptive statistics for the SDQ scores.

	CPU Mean (*SD*)	Control Mean (*SD*)
Overall Difficulties T0	8.58 (7.49)	7.33 (5.41)
Overall Difficulties T1	6.82 (6.55)	8.01 (6.27)
Overall Difficulties T2	8.17 (7.24)	8.43 (6.67)
Externalizing Problems T0	5.03 (5.28)	3.93 (3.71)
Externalizing Problems T1	4.09 (4.84)	4.52 (4.29)
Externalizing Problems T2	4.67 (5.19)	4.81 (4.22)
Internalizing Problems T0	3.55 (3.44)	3.40 (2.83)
Internalizing Problems T1	2.73 (2.99)	3.49 (3.04)
Internalizing Problems T2	3.50 (3.61)	3.62 (3.35)
Prosocial Behavior T0	7.06 (2.47)	7.05 (2.29)
Prosocial Behavior T1	7.90 (2.04)	7.01 (2.56)
Prosocial Behavior T2	7.72 (2.47)	7.21 (2.56)

**Table 2:** Mixed models predicting changes in the SDQ scales.

	Overall		Externalizing		Internalizing		Prosocial	
	Est. (*S.E.)*	C.I.	Est. (*S.E.)*	C.I.	Est. (*S.E.)*	C.I.	Est. (*S.E.)*	*C.I.*
Intercept	6.87 (0.68)	5.45–8.28	3.54 (0.042)	2.67–4.41	3.31 (0.37)	2.55–4.07	7.00 (0.25)	6.48–7.52
Group	2.09 (0.96)	0.10–4.08	1.80 (0.58)	0.59–3.02	0.32 (0.51)	−0.73–1.38	−0.27 (0.35)	−1.01–0.46
Time	0.57 (0.20)	0.17–0.97	0.44 (0.12)	0.21–0.68	0.12 (0.12)	−0.11–0.35	0.02 (0.08)	−0.14–0.19
Group × Time	−1.37 (0.28)	−1.93–−0.82	−0.91 (0.16)	−1.23–−0.58	−0.42 (0.16)	−0.74–−0.098	0.43 (0.12)	0.20–0.66
Subjects: Random Intercept	34.32 (3.07)	28.79–40.91	18.59 (1.61)	15.68–22.04	7.04 (0.67)	5.83–8.50	4.16 (0.39)	3.44–5.02
Classrooms: Random Intercept	0.92 (1.04)	0.10–8.4	0.096 (0.39)	0.00–303.59	0.35 (0.27)	0.073–1.64	0.12 (0.13)	0.01–1.01

*Note.* Est. = estimate; S.E. = standard error; C.I. = 95% confidence interval. Statistically significant results (*p* < 0.05) are in boldface.

## Data Availability

The data presented in this study are available on request from the corresponding author.
